# Supplementary material for: Chromothripsis during telomere crisis is independent of NHEJ, and consistent with a replicative origin
Source: Genome Res. 2019 May;29(5):737–49. doi: 10.1101/gr.240705.118 (PMC6499312; doi:10.1101/gr.240705.118)
Supplement: Supplemental Material [file supp_gr.240705.118_Supplemental_file_1.zip › contigs/annotated_contigs/DB93/contig.2.DB93_length_397_mean_cov_6.29722921914.docx]

**DB93_length_397_mean_cov_6.29722921914**

```
TAGATAGATCGATCGATAGATAGAGATAGATGATAGATCGATAAATGGGAGCTTATTAAATATTAACTGACATGATCACAAGGCTCCAC
 >chr18:1333120-1333231 - E=3e-46
AACAGGCTGTGTGCAAGCTGA|G|ATTCAATGAATTTTACTTCTCTCATTCTTCAGACTGATATATTAGAATGTCCTAGATGCCACAGA
                      >chr18:3081604-3081891 + E=2e-155
GGAATTTCCTGGAATTTGATCTATTTTAGCTAATAATTACTTTCCTAAAATGTATTCTGAAAGAAACAGAACAAAGGAATAAGCCCCAA

GCATTCTACATTGGAATTTTACTAATTCTCATTTTTATGACTCAGGAAATAATCAATCATAATACTAACGAAAACACTGTTTTCTATAA

CAGTAAAGTCATCTTAAAGAAAACACAAATGGCTGCTTTGTGG
```
